# Severe pulmonary hypertension in chronic obstructive pulmonary disease – From clinical perspective to histological evidence

**DOI:** 10.1016/j.ijcchd.2024.100519

**Published:** 2024-06-12

**Authors:** Katarina Zeder, Teresa Sassmann, Vasile Foris, Philipp Douschan, Horst Olschewski, Gabor Kovacs

**Affiliations:** a Division of Pulmonology, Medical University of Graz, Austria; b Ludwig Boltzmann Institute for Lung Vascular Research Graz, Austria; c Division of Cardiovascular Medicine, University of Maryland School of Medicine, Baltimore, MD, USA; d University of Maryland-Institute for Health Computing, Bethesda, MD, USA; e Channing Division of Network Medicine, Department of Medicine, Brigham and Women's Hospital and Harvard Medical School, Boston, MA, USA

**Keywords:** Pulmonary hypertension, Chronic obstructive pulmonary disease, Remodeling, Diagnosis, Survival

## Abstract

Severe pulmonary hypertension (PH) in chronic obstructive pulmonary disease (COPD) is currently defined by an elevated mean pulmonary arterial pressure and strongly elevated pulmonary vascular resistance >5 wood units. Clinically, these patients show a male predominance, and usually present with very severe dyspnea, severe hypoxemia, strongly decreased exercise capacity and poor prognosis, even though the clinical picture is frequently associated with less severe airflow obstruction. Explanted lung samples of patients with COPD and severe PH show severe remodeling of small pulmonary arterioles, predominantly in the intima and media of the vessels. In this concise review, we discuss the clinical and histopathological evidence of severe PH in COPD.

## Abbreviations

%pred: per cent predicted
6MWD: 6-min-walking distance
Ao: aorta
ASPIRE: Assessing the Severity of Pulmonary Hypertension In a Pulmonary Hypertension REferral Centre
BMI: body mass index
BSA: body surface area
COMPERA: Comparative, Prospective Registry of Newly Initiated Therapies for Pulmonary Hypertension
COPD: chronic obstructive pulmonary disease
DLCO: single breath diffusing capacity of lung for carbon monoxide corrected for haemoglobin
FEV1: forced expiratory volume in 1 s
FVC: forced vital capacity
GOLD: Global Initiative For Chronic Obstructive Lung Disease
HE: hematoxylin eosin staining
HES: heath edwards scale
ILD: interstitial lung disease
IPAH: idiopathic pulmonary arterial hypertension
mPAP: mean pulmonary arterial pressure
NT-pro-BNP: N-terminal pro brain natriuretic peptide
PA:Ao Ratio: ratio from pulmonary artery to aorta
PAH: pulmonary arterial hypertension
PAS: periodic acid shiff staining
PAWP: pulmonary arterial wedge pressure
peak VO2: maximal oxygen uptake
PH: pulmonary hypertension
PVD: pulmonary vascular disease
PVR: pulmonary vascular resistance
RHC: right heart catheterization
SMA: smooth muscle actin staining
SR: sirius red staining
TAPSE: tricuspid annular plane systolic excursion
TC: trichrome staining
TLC: total lung capacity
V/Q: ventilation/perfusion
WHO FC: world health organization functional class
WSPH: World Symposium on Pulmonary Hypertension
WU: wood units

## 1 Introduction

Pulmonary hypertension (PH) associated with chronic obstructive pulmonary disease (COPD) is classified as World Health Organization (WHO) group 3 PH [1]. In Europe and North-America, group 3 PH accounts for around 48 % of all PH cases and is thus a highly prevalent finding [2], especially considering an estimated global prevalence of all-cause PH of 1 % [1]. When present, PH in COPD is associated with decreased exercise capacity, quality of life and survival. A subgroup of patients develops severe PH, which is associated with severe dyspnea, severe hypoxemia, strongly decreased exercise capacity and very poor prognosis despite usually presenting with less severe airflow obstruction and emphysema as compared to patients with COPD but only mild PH or no PH. In spite of the increasing awareness for pulmonary vascular involvement in COPD and growing clinical experience, there are many gaps in current knowledge. Below, we identify some of the most actual questions and try to answer them based on the currently available evidence.

## 2 What is severe PH in COPD and how is it defined?

The term “severe PH in COPD” was first introduced by the 5th World Symposium on Pulmonary Hypertension (WSPH) and defined as mean pulmonary arterial pressure (mPAP) ≥ 35 mmHg or mPAP ≥25 mmHg in combination with a cardiac index <2 L/min/m^2^ [3]. The 2015 ESC/ERS PH Guidelines [4] and the 6th WSPH [5] adopted this definition. The definition, however, was changed in the 2022 ESC/ERS PH Guidelines [1]. Accordingly, PH in COPD may be non-severe (mean pulmonary arterial pressure (mPAP) > 20 mmHg and pulmonary vascular resistance (PVR) ≤ 5 Wood units (WU)) or severe (mPAP >20 mmHg and PVR >5 WU) [1]. The introduction of the PVR >5 WU threshold to define severe PH is mainly based on two retrospective analyses that demonstrated that in patients with COPD and interstitial lung disease (ILD), a strongly elevated PVR was a more robust predictor of poor prognosis than mPAP [6,7].

**Fig. 1:**
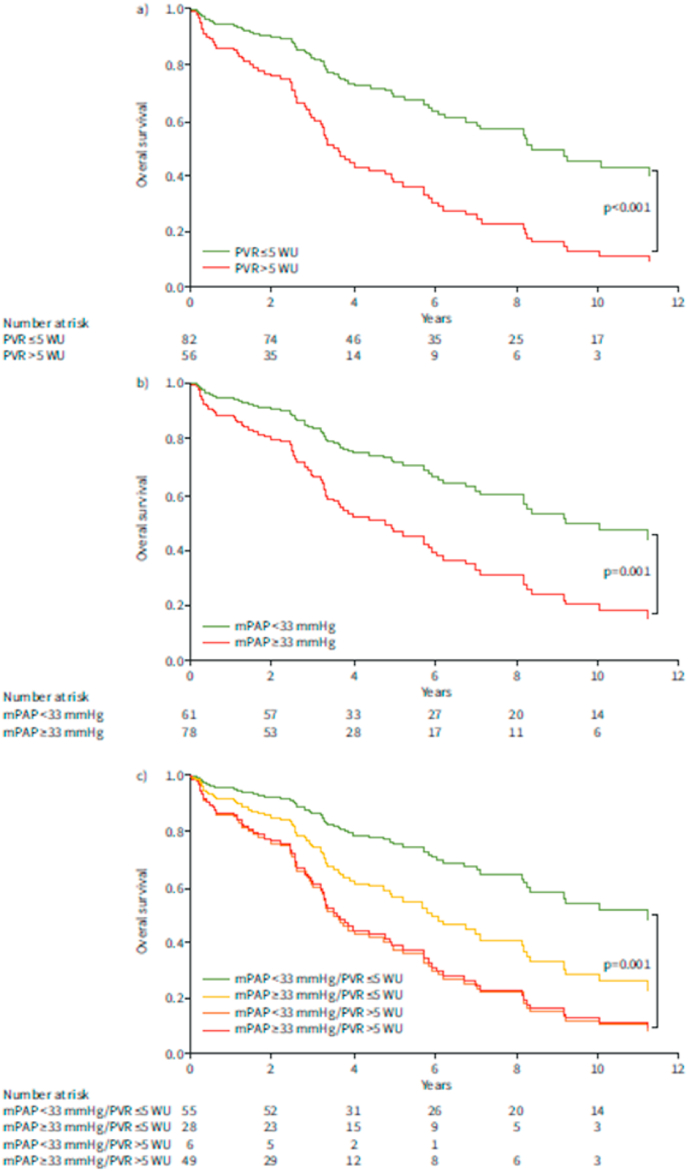
a) Multivariate Cox regression analysis by pulmonary vascular resistance (PVR) groups (cut-off: PVR >5.0 WU) accounting for age, sex and forced expiratory volume in 1 s (FEV1) (n = 138; HR 2.59, 95 % CI 1.58–4.27; p < 0.001). b) Multivariate Cox regression analysis by mean pulmonary arterial pressure (mPAP) groups (cut-off: mPAP ⩾33 mmHg) accounting for age, sex and FEV1 (n = 139; HR 2.26, 95 % CI 1.37–3.71; p = 0.001). c) Univariate Cox regression analysis by mPAP and PVR groups with mPAP cut-off ⩾33 mmHg and PVR cut-off >5.0 WU (n = 138; p = 0.001; reference category: mPAP <33 mmHg and PVR ⩽5.0 WU (green curve); versus mPAP ⩾33 mmHg and PVR ⩽5.0 WU (yellow curve): HR 2.02, 95 % CI 1.04–3.93; versus mPAP <33 mmHg and PVR >5.0 WU (orange curve): HR 3.45, 95 % CI 1.25–9.52; versus mPAP ⩾33 mmHg and PVR >5.0 WU (red curve): HR 3.35, 95 % CI 1.95–6.06). For data in panels a and c, n = 138 as pulmonary arterial wedge pressure and therefore PVR was not available in one patient. Reproduced with permission of the ERS 2024: European Respiratory Journal 58 (2) 2100944; **DOI:** 10.1183/13993003.00944-2021 Published 26 August 2021

In the first study, 139 COPD patients (55 % males, median age 68 years) were analyzed who had undergone right heart catheterization (RHC) at the Medical University of Graz [7]. During a median follow-up of 8 years, 52 % of the patients died, the three main leading causes of death being COPD (35 %), cancer (18 %) and heart failure (18 %). 44 % of patients received at least one PH drug during follow-up [7]. Out of the available pulmonary hemodynamic variables, both PVR (HR: 1.09 [1.02–1.16], p = 0.007) and mPAP (HR: 1.03 [1.01–1.05], p = 0.001) were significantly associated with increased mortality, but the PVR >5.0 WU cut-off turned out to be the best hemodynamic threshold to predict survival (HR: 2.59 [95 % CI 1.58–4.27], p < 0.001; Fig. 1) [7].

The second study analyzed 449 patients from the Comparative, Prospective Registry of Newly Initiated Therapies for Pulmonary Hypertension (COMPERA) registry who were classified as having PH associated with ILD. A PVR >5 WU threshold identified patients with a significantly worse survival as compared to PVR ⩽5 WU (p = 0.03), while the mPAP thresholds from the previous definition of severe PH did not identify patients with poor prognosis (p = 0.18) [6].

Of note, PVR has been frequently considered as key hemodynamic variable for pulmonary vascular disease, better reflecting the severity of pulmonary vascular remodeling than mPAP. It was a strong prognostic parameter in a general patient population of over 40,000 US Veterans [8] and its prognostic relevance has been previously described in patients with ILD and COPD [9]. In addition, in a recent publication from the COMPERA registry, PVR was a significant independent predictor of transplant-free survival in over 370 COPD patients with PH [10]. The elevation of mPAP may be less specific for pulmonary vascular remodeling in lung diseases than PVR elevation. This may be due to the fact that mPAP is significantly increased not only due to vascular remodeling but also due to post-capillary PH in case of left heart disease and intrathoracic pressure increase in case of severe emphysema [7,11]. Nevertheless, an elevation in mPAP has also emerged as robust variable for predicting poor outcome in COPD. Data from the ASPIRE registry revealed that COPD patients with mPAP ≥40 mmHg had impaired prognosis as compared to COPD patients with less severe mPAP elevation [12]. In fact, with a 1-year survival of 70 % and 3-year survival of 33 %, COPD patients with severely elevated mPAP belong to the PH patients with the poorest prognosis [10].

## 3 How much do airflow obstruction and vascular obstruction contribute to increased mortality in COPD?

**Fig. 2:**
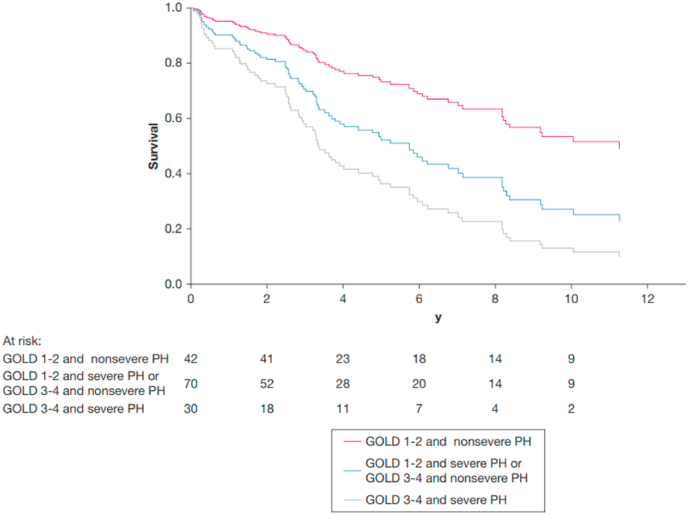
Cox-regression survival analysis for patients with COPD based on severity of airflow limitation and PH. Red curve indicates good prognosis, consisting of GOLD stages 1 or 2 and no or moderate PH. Blue curve indicates intermediate prognosis, including either GOLD stages 3 or 4 or severe PH. Gray curve indicates poor prognosis, with the combination of GOLD stages 3 or 4 and severe PH. ©Chest. Kovacs et al., 2022, https://doi.org/10.1016/j.chest.2022.01.031page 208, Fig. 2), reprinted with permission.

A recent retrospective study from our center investigated predictors of mortality in a cohort of well characterized COPD patients who underwent RHC due to suspicion of significant PH [13]. When combining GOLD stages and PH severity (non-severe vs severe PH), three distinct survival groups were identified (Fig. 2): patients with the best survival (3-year survival: 90 %) had GOLD stage 1 or 2 and non-severe PH; patients with intermediate survival (3-year survival: 68 %) had GOLD stages 3 or 4 in combination with non-severe PH or GOLD stages 1 or 2 in combination with severe PH; and patients with the worst survival (3-year survival: 54 %) had a combination of GOLD 3 or 4 and severe PH [13]. These data suggest that in patients with PH and COPD, both the severity of airflow limitation and pulmonary vascular disease equally and independently contribute to increased mortality.

## 4 How can we identify patients with COPD and severe PH?

The clinical presentation of PH in COPD is unspecific, as both conditions present with dyspnea on exertion as their main feature [1]. Several non-invasive tools may suggest the presence of PH in COPD patients, including laboratory testing (especially N-terminal pro-brain natriuretic peptide (NT-pro-BNP), diffusion capacity for carbon monoxide (DLCO), echocardiography, chest CT, 6-min walk test, cardiopulmonary exercise testing and electrocardiogram (ECG) [5]. However, none of these tools has a sufficiently high sensitivity and specificity, when used in isolation. Elevated levels of NT-pro-BNP may be indicative for PH associated with COPD, however, natriuretic peptide levels may be also increased in the presence of left heart disease, which is a common comorbidity in COPD patients [14]. DLCO values are frequently very low, when significant PH is present, but this is quite unspecific because it may also be low due to severe emphysema or accompanying fibrotic changes. ECG abnormalities, like the occurrence of a P pulmonale, right axis deviation, RV hypertrophy, RV strain, right bundle branch block, and QTc prolongation may substantiate the suspicion of right ventricular burden due to PH. Nevertheless, a normal ECG does not exclude PH [4]. In chest CT, the ratio of the diameters of the main pulmonary artery to the ascending aorta >1 was associated with PH in COPD [5,15]. Finally, when assessing physical capacity via 6-min walk test or cardiopulmonary exercise testing, COPD patients with PH typically show poorer exercise capacity and more impaired gas exchange than COPD patients without PH [5]. Beyond all these tools, in clinical practice, echocardiography is considered as the best non-invasive method to suspect PH. The tricuspid regurgitation velocity (TRV) may be used to estimate the systolic pulmonary arterial pressure, where a peak TRV >3.4 m/s is associated with a high probability of PH [1]. In case of COPD patients, other signs of PH, including right ventricular and right atrial enlargement, pericardial effusion, enlargement of the inferior vena cava and a decreased right ventricular function measured by reduced tricuspid annular plane systolic excursion, are often applied to estimate the likelihood of PH [1], particularly when severe emphysema limits the assessment of TRV [16]. Besides its advantages, especially in patients with chronic lung diseases, echocardiography suffers from low precision, which limits its diagnostic power on the individual level.

Due to the mentioned limitations of diagnostic tools, the combination of non-invasive methods appears promising for the prediction of PH and severe PH in patients with COPD. In a recent large retrospective analysis, the combination of estimated sPAP ≥56 mmHg from echocardiography, NT-pro-BNP ≥650 pg/ml and the pulmonary artery to ascending aorta diameter ratio ≥0.93 in chest CT was quite strongly associated with severe PH. This combination of variables predicted severe PH with a high sensitivity and specificity (94 % and 95 %, respectively). Of note, if none of the above criteria was present, the probability of severe PH was only 7 % [13].

The gold standard for the diagnosis of PH remains RHC, although is an invasive method. According to current guidelines, RHC should only be performed in COPD patients, when this may result in a change in the clinical management [5]. This includes referral for lung transplantation, inclusion in clinical trials, treatment of unmasked left heart disease or an individualized treatment approach in the presence of severe PH [5].

## 5 How can we distinguish between severe PH associated with COPD (group 3 PH) vs. severe PAH with COPD as comorbidity (group 1 PH)?

It is not always easy to decide, if a patient should be considered as PAH with concomitant COPD (group 1) or PH associated with COPD (group 3). This decision is, however, of great relevance, since this may decide whether a COPD patient is considered to be a candidate for targeted PAH therapy or not [17]. Some help for differentiation has been provided by the current guidelines, acknowledging the fact, that the spectrum of severity of the vascular disease and the parenchymal disease represent a continuum and it is often difficult to define clear thresholds. Currently used criteria for the decision if a patient should be classified as group 1 or group 3 PH are based on pulmonary function tests, high-resolution chest CT, hemodynamic profile in RHC and cardiopulmonary exercise testing [5]. As an example, in patients with mild airflow limitation, mild or missing lung parenchymal abnormalities in the CT scan and a significant cardiovascular limitation in cardiopulmonary exercise testing, group 1 will be favored. In contrast, in patients with more severe airflow limitation, significant abnormalities in chest high resolution CT scan and a predominant ventilatory limitation in the cardiopulmonary exercise testing will favor group 3 PH [3]. Due to the complexity and often overlapping features characterizing patients with PH and COPD, classification is challenging and decisions on their management should only be made by physicians experienced in both PH and COPD.

## 6 The “pulmonary vascular phenotype” in COPD?

“Pulmonary vascular phenotype” is a term that has been suggested for a small group (around 1 %) of COPD patients with moderate airflow limitation, but severe pre-capillary PH, no or very mild hypercapnia, very low DLCO (<45 % predicted), circulatory exercise limitation and poor prognosis [18]. Although patients with the “pulmonary vascular phenotype” should be considered as group 3 PH, they share many hemodynamic and clinical characteristics with group 1 PH (pulmonary arterial hypertension) patients.

## 7 The “lung phenotype” in IPAH?

The use of this term has resulted from an analysis of the COMPERA registry that identified three clusters of IPAH patients based on their age, sex, comorbidities, smoking status and DLCO [19]. The largest of the clusters, containing nearly 50 % of the patients, consisted mainly of elderly males, who were previous or current smokers and presented with a high rate of cardiovascular comorbidities. DLCO was strongly reduced in many of these subjects and survival was poor, despite PH therapy [19]. In a validation study, the term “IPAH lung phenotype” has been introduced for IPAH patients who were former or current smokers and had a low DLCO (<45 % predicted) and their responses to PAH therapy and their prognosis were analyzed in both the COMPERA and ASPIRE registry [20]. These patients had little in common with classical IPAH patients, who have been enrolled in all of the pivotal PAH studies, characterized by relatively young age, female preponderance and very few comorbidities. Similar to those with classical IPAH, the IPAH lung phenotype patients presented with severe pre-capillary PH, but all other characteristics were very similar to group 3 PH patients, including poor response to PAH drugs and devastating survival [20].

It is important to state that despite their similarities, the COPD pulmonary vascular phenotype and the IPAH lung phenotype are not the same. COPD patients with the pulmonary vascular phenotype are characterized by a moderate airflow limitation, whereas most of the patients with the IPAH lung phenotype had normal or near normal lung function tests and the majority of them had no or only mild parenchymal involvement [20].

## 8 Should we treat COPD patients associated with severe PH (group 3) with PAH medications?

In principle, treatment with PAH drugs has not been recommended for group 3 patients [1], because to date there are no sufficiently powered prospective randomized controlled trials showing a benefit of such treatment. The recently published PERFECT study was the largest prospective RCT in PH associated with COPD and showed no improvement in the primary efficacy end point after inhaled treprostinil as compared to placebo (change in peak 6-min walk distance (6MWD) after 12 weeks was −4.5 vs. −5.1 m in the treatment vs. placebo group). In addition, patients treated with inhaled treprostinil experienced numerically more adverse events, serious adverse events, deaths, treatment discontinuations, and study discontinuations. Therefore, the study has been terminated as recommended by the Data and Safety Monitoring Committee [21].

However, retrospective studies and some smaller prospective trials suggested that PAH drugs might be beneficial in some patients. Accordingly, the current ESC/ERS PH guideline suggest an individualized approach to treatment for patients with COPD and severe PH [1]. In contrast, PAH drugs have been discouraged for COPD patients with non-severe PH [1]. Of note, in some of the patients, PAH medications may also deteriorate exercise capacity, gas exchange and outcomes and should therefore always be used with caution and only in expert centers that provide close follow-up intervals [1].

## 9 Is there histological evidence for severe PH in COPD?

Our recent study systematically investigated remodeling of the small pulmonary arteries, parenchyma and small airways in explant lungs from end-stage COPD patients with and without PH, as compared to donor lungs and IPAH lungs, providing the first compartment specific histological analysis in COPD patients. The main objective was to compare age-and sex-matched samples of mild vs. severe PH of end-stage COPD patients. The degree of remodeling of the three compartments, vessels, airways, and parenchyma showed significant differences between COPD patients with severe vs. mild PH, and also between COPD patients vs. IPAH patients or donor lungs [22].

**Fig. 3:**
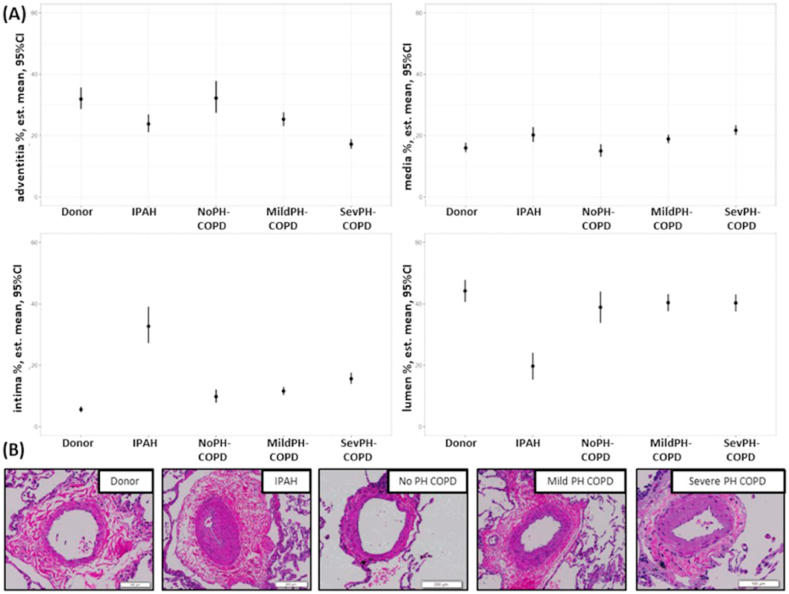
Comparison of small pulmonary arterial remodeling between donors, IPAH, noPH COPD, mildPH COPD, and sevPH COPD. (A) Systematic analysis of all orthogonal cuts of pulmonary arteries and arterioles <200 μm of all probes. (B) Representative pulmonary arteries and arterioles in the different groups (hematoxylin-eosin staining). For significant differences between groups please see Table 2. COPD, chronic obstructive pulmonary disease; IPAH, idiopathic PAH; MildPH-COPD, COPD with mild PH; NoPH-COPD, COPD without PH; SevPH-COPD, COPD with severe PH.

In the vascular compartment, COPD patients with severe PH had a higher degree of intimal thickening as compared to COPD patients with mild or no PH or donors, although this intimal thickening was still much milder than in patients with IPAH. In addition, medial thickening was also very prominent in COPD patients with severe PH (Fig. 3). Of note, significant pulmonary arterial remodeling was already found in COPD patients with no PH (Fig. 3), which may be suggestive of vascular remodeling preceding hemodynamic changes [22]. Similar findings have been published in patients with pulmonary veno-occlusive disease and in systemic sclerosis [23,24]. Intimal thickening has also been induced by cigarette smoking, as demonstrated in the smoking mouse model [25], in smokers without airflow obstruction [26] and in the guinea pig model of COPD [27]. These observations suggest a direct effect of cigarette smoke on the pulmonary endothelium, and there is strong evidence for the involvement of bone-marrow derived cells [28]. Nevertheless, the complex mechanisms underlying the different remodeling patterns remain poorly understood.

Remodeling of small pulmonary arteries in transplanted lungs of end-stage COPD patients has been investigated in two further studies [29,30]. Both studies emphasize the different remodeling pattern in COPD with severe PH as compared to COPD with mild or no PH [29,30]. The first study graded vascular lesions according to a modified Heath and Edwards scale (HES) from 1 to 6 [30], a scale which has been introduced to describe histologic lesions in IPAH. The authors observed a correlation between the extent of pulmonary vascular lesions with the severity of PH. Additionally, morphologic lesions, similar to those characteristic of IPAH, were found in COPD with severe PH only [30]. In the second study, the thickness of the wall of medium-sized arteries, the remodeling of microvessels, and the pulmonary capillary density have been analyzed. In patients with COPD and severe PH, the remodeling score of the microvessels was significantly higher (p < 0.0045) and the capillary density was lower (p < 0.005) than in COPD patients with mild PH [29].

**Fig. 4:**
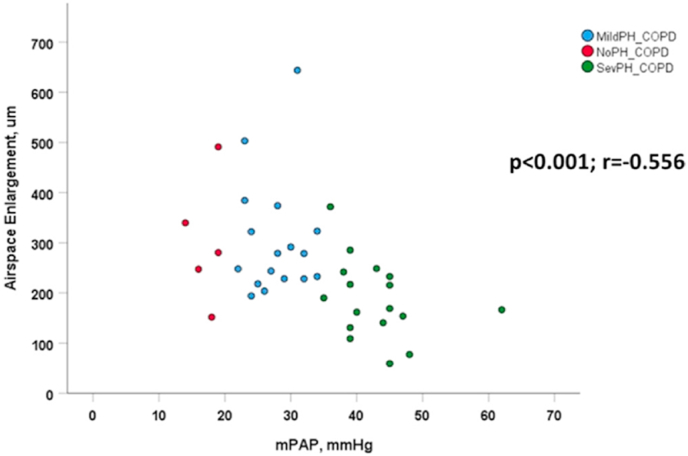
Correlation between mean septal distance and mPAP in patients with COPD with no PH, mild PH, and severe PH. The significant negative Pearson correlation (r = −0.556) suggests that a higher degree of pulmonary vascular remodeling is associated with a lesser degree of lung emphysema (p < 0.001). mPAP, mean pulmonary arterial pressure; MildPH-COPD, COPD with mild PH; NoPH-COPD, COPD without PH; SevPH-COPD, COPD with severe PH.

Regarding the parenchymal compartment, the mean interseptal distance may be used as surrogate for the degree of emphysema [22]. In our study, mean interseptal distance was most increased in COPD patients without PH, while COPD patients with mild PH and severe PH showed less and less emphysema. As expected, DLCO was negatively correlated with mean interseptal distance. Accordingly, there was an inverse relationship between emphysema severity as assessed by histology and pulmonary arterial pressure [22] (Fig. 4). This suggests that DLCO is stronger related to emphysema than to pulmonary hypertension and that vascular remodeling is not directly related to parenchymal remodeling [22]. This finding is consistent with findings from a CT study demonstrating that emphysema is less prominent in COPD patients with severe PH [31]. The analysis of the airways showed a tendency towards less mucus-producing cells and less CD45^+^ cells in the airway epithelium in COPD patients with severe PH as compared to mild PH [22], which might be suggestive of less severe airway remodeling and inflammation in COPD with severe PH, reflecting the parenchymal changes.

## 10 Conclusion

**Fig. 5:**
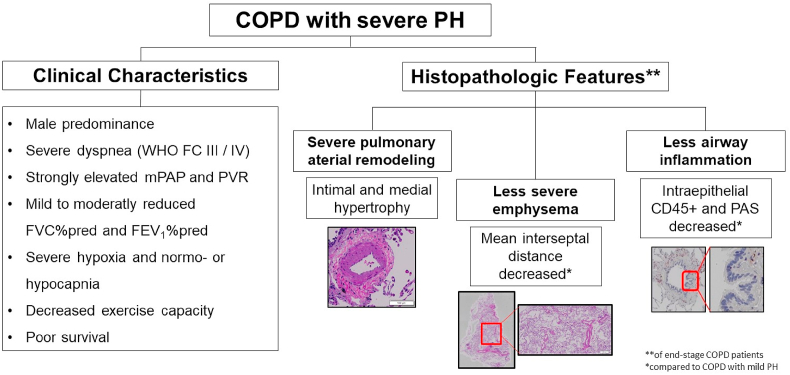
Central illustration of COPD patients with Severe PH, demonstrating the clinical and histopathologic changes in COPD patients with severe PH. PAS: Periodic Acid Shiff, FEV1: Forced Expiratory Volume in 1 s, WHO FC: World Health Organization Functional Class, mPAP: mean pulmonary arterial pressure, PVR: pulmonary vascular resistance, FVC: Forced Vital Capacity.

Severe pulmonary hypertension (PH) in chronic obstructive pulmonary disease (COPD) is defined by an elevated pulmonary arterial pressure and pulmonary vascular resistance (PVR) > 5 wood units. Clinically, these patients present with a male predominance, severe dyspnea, severe hypoxemia, strongly decreased exercise capacity and poor prognosis despite less severe airflow obstruction and degree of emphysema (Fig. 5). Although data from prospective randomized controlled trials are missing, individualized treatment may be considered for severe PH in expert centers with experience in the treatment of both COPD and PAH. Histologic correlates of severe PH in transplanted lung tissue samples from end-stage COPD patients show severe remodeling of small pulmonary arterioles, predominantly in the intima and media of the vessels. The histologic assessment of emphysema suggests less severe emphysema in more severe PH and vice versa.

## Financial support

none.

## CRediT authorship contribution statement

**Katarina Zeder:** Writing – review & editing, Writing – original draft, Data curation, Conceptualization. **Teresa Sassmann:** Writing – review & editing. **Vasile Foris:** Writing – review & editing. **Philipp Douschan:** Writing – review & editing. **Horst Olschewski:** Writing – review & editing. **Gabor Kovacs:** Writing – review & editing, Supervision, Conceptualization.

## Declaration of Competing interest

There is no coflict of interest.
